# Recognition and Management of Button Battery Ingestion Amongst Emergency Practitioners

**DOI:** 10.7759/cureus.18929

**Published:** 2021-10-20

**Authors:** Adnan Darr, Zahir Mughal, Somiah Siddiq

**Affiliations:** 1 Otolaryngology, The Royal Wolverhampton NHS Trust, Birmingham, GBR; 2 Otolaryngology, South Warwickshire NHS Foundation Trust, Warwick, GBR; 3 Otolaryngology, University Hospitals Birmingham NHS Foundation Trust, Birmingham, GBR

**Keywords:** pediatric endoscopy, pediatric emergency department (ped), patient safety, esophageal foreign body, swallowed foreign body, button battery

## Abstract

Introduction

Button battery ingestion (BBI) carries a significant risk of morbidity and mortality. We conducted a regional analysis following an NHS England national patient safety alert to evaluate the knowledge base in the emergency management of BBI amongst emergency practitioners.

Methods

A ten-point questionnaire was distributed electronically and in hardcopy formats to emergency departments across 15 hospitals within the West Midlands, United Kingdom. The questionnaire assessed participants’ knowledge of emergency management of BBI. The effect of clinician grade and previous otorhinolaryngology experience on knowledge scores was evaluated.

Results

A total of 176 responses were received from 11 hospitals. A small proportion (18%) were aware of a local department protocol. The majority of participants (70%) routinely screened for a button battery in their history taking. Our findings highlighted a lack of awareness of the timeframe for mucosal injury, potential complications, radiological signs, and the necessity for immediate retrieval. The median knowledge score was 18.8% (IQR=12.5-31.3%). Both registrars and consultants scored the highest (median 25%). Previous otorhinolaryngology experience was associated with a higher median score (P=0.002).

Conclusion

Our multi-center regional emergency medicine analysis demonstrated knowledge deficiency in the initial assessment and management of BBI. A high index of suspicion for button battery ingestion is needed. In view of the time-critical nature of button battery impaction in the esophagus, a “golden hour” concept should be integrated into acute management pathways with the early involvement of otorhinolaryngologists.

## Introduction

Button batteries are used to power a considerable number of electronic items in the household. A large series of over 8,000 button battery ingestion (BBI) cases identified the top four offending objects were hearing aids (36%), electronic games and toys (22%), watches (11%), and calculators (6%) [1]. The signs and symptoms of BBI in the upper aerodigestive tract are variable and non-specific, ranging from an asymptomatic to a clinically unstable patient. Non-specific symptoms include pain, cough, emesis, irritability, fever, and tachycardia, whilst more specific symptoms include drooling and reduced oral intake [2]. The clinical evaluation can be challenging, especially in the very young, with misdiagnosis and subsequent delay in treatment as high as 54%, particularly given a significant proportion tend to be unwitnessed ingestions (46%). Button battery impaction in the esophagus has the potential to cause serious morbidity as early as two hours [3]. Complications are related to the propensity of button batteries to cause caustic injury by hydrolysis of water and generation of hydroxide ions; this leads to alkaline-induced liquefactive necrosis [4]. Early complications include severe burns, hemorrhage, visceral perforation, and airway obstruction; late complications include fistula formation, esophageal stricture/stenosis, and vocal cord paralysis [3].

The widespread use of button batteries within households, the challenges encountered in assessing such patients, and the short interval before catastrophic complications develop all combine to create a time-critical emergency for frontline emergency department (ED) providers. All providers should therefore be knowledgeable in the signs, symptoms, complications, investigations, and management of BBI [4]. Following several national incidents of missed BBI, NHS England declared a national patient safety alert, which took effect in January 2015. NHS organizations were instructed to implement local protocols aimed at prompt recognition and immediate removal of button batteries [5]. We aimed to assess ED practitioners’ awareness of local hospital protocols relating to BBI and to evaluate knowledge relating to the initial assessment and management of patients presenting acutely. The effect of previous otorhinolaryngology experience and clinician grade on knowledge was also evaluated.

## Materials and methods

A ten-point questionnaire was designed in-house by the co-author Adnan Darr (AD) and disseminated to ED practitioners across 15 hospitals within the West Midlands, United Kingdom (UK). Distribution platforms included internal and electronic mail. Of the 15 hospitals, one was a tertiary pediatric referral center, whilst the other units delivered both pediatric and adult emergency services. Data was captured over a 12-week period. The survey sought to determine demographic data, the existence of departmental policies, and targeted knowledge on the assessment and management of patients with suspected BBI. The survey is provided in Table 1. The responses were used to deduce a score for knowledge. The maximum attainable score was 16 for correct responses in questions relating to the time frame (one mark), complications (eight marks), the interval for intervention (one mark), investigations (four marks), and radiological signs (two marks). Statistical analysis was performed using Microsoft Excel 2019 (Microsoft, Redmon, Washington) and GraphPad Prism 5 (GraphPad, San Diego, California, US). Knowledge scores were analyzed to produce median and interquartile ranges (IQR). The effect of clinician grade on scores was assessed using the Kruskal-Wallis test with Dunn’s post hoc adjustment for multiple comparison testing. The difference in scores between clinicians with and without prior otorhinolaryngology posts was assessed using the Mann-Whitney U test.

**Table 1:** Button battery ingestion questionnaire FY1/2 = foundation year 1/2, SHO = senior house officer, NCEPOD = National Confidential Enquiry into Patient Outcome and Death

Button battery survey:	
1) Name of the current hospital?	
2) Your grade:	FY1/FY2/SHO/Registrar/Middle grade/Cons
3) Have you previously held a post in otorhinolaryngology?	Yes / No
4) Is there a local protocol for ingested button batteries at your hospital?	Yes / No / Unsure
5) Do you routinely screen for button batteries in suspected foreign bodies ingestion?	Yes / No
6) Button batteries may cause tissue damage as early as:	1 hr / 2 hrs / 4 hrs / 8 hrs / >12 hrs
7) List as many complications of an ingested button battery in the esophagus:	
8) How soon must an ingested button battery be removed from the esophagus?	Minutes (NCEPOD 1): Immediate
	Hours (NCEPOD 2): Urgent
	2 days (NCEPOD 3): Expedited
	Elective (NCEPOD 4): Elective
9) What discriminating investigation(s) would you request if you are suspecting an ingested button battery?	
10) In the above investigation(s), what features would discriminate between a button battery and an object of similar appearance?	

## Results

**Figure 1:**
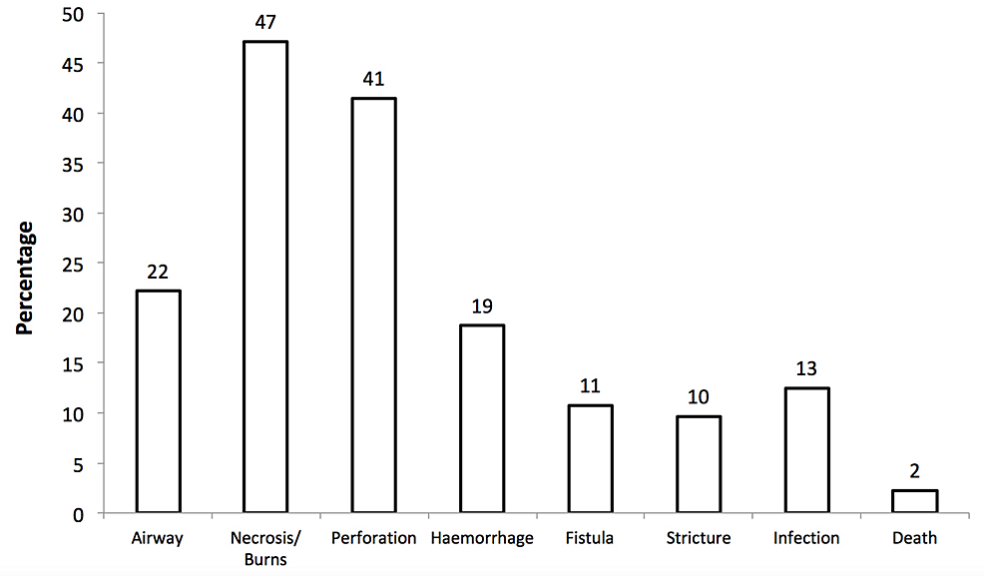
The proportion of participants that named specific complications of button battery ingestion when asked to list as many as possible in the questionnaire

A total of 176 responses were collected from 11 emergency departments. Most participants were senior house officers (SHO) (39%), followed by foundation year 2 (FY2) trainees (26%), registrars (21%), consultants (10%), and foundation year 1 (FY1) trainees (4%). Of the respondents, 18% had previously held an otorhinolaryngology post during their post-graduate training. Of the 176 respondents, 18% (n=32) were aware of an internal protocol for BBI. Of the remaining 82%, 27% stated no local protocol existed, and 73% did not know. Nearly 70% (n=123) stated that they routinely screened for BBI in their history taking when assessing a patient with suspected foreign body ingestion. Twenty-seven percent (n=48) of respondents correctly identified that mucosal damage would occur as early as two hours. Of the remaining respondents, 35% (n=61) stated one hour; 29% (n=51) stated four hours; 4.5% (n=8) stated eight hours; and 4.5% (n=8) stated 12 hours. One in seven participants (n=25) were unable to list a single complication, whilst others stated necrosis/localized mucosal burns (47%, n=83), perforation (41%, n=72), and airway obstruction (22%, n=39) (Figure 1). The total number of complications listed per respondent was a median of two (IQR=1-2). Thirteen percent (n=23) correctly identified the need for a combination of neck, chest, and abdominal plain film radiographs, and 43% (n=10) also identified the necessity for lateral views. The other responses comprised of only neck (28%, n=49), chest (60%, n=106), or abdominal (28%, n=49) radiographs. Typical radiological features of BBI such as “halo” and “step-off” signs were stated by 13% (n=23) and 10% (n=18), respectively, and both signs were recognized by 4% (n=7) of respondents. Time to surgery, as defined by the National Confidential Enquiry into Perioperative Deaths (NCEPOD) [6], was prioritized correctly as immediate by 25% (n=44) of participants. Others selected urgent (72%, n=127), expedited (2%, n=4), and elective (1%, n=1). 

**Figure 2:**
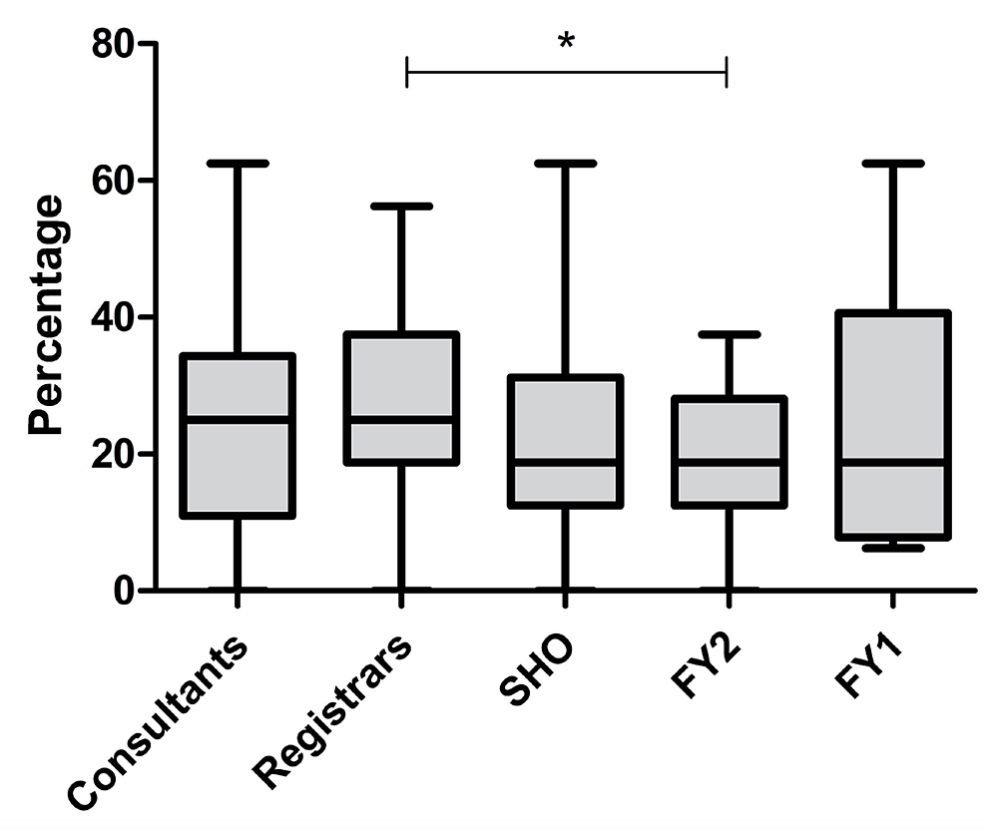
Box and whisker plot showing percentage scores on the knowledge-based questionnaire Upper and lower limits of the boxes indicate upper and lower quartiles, respectively; horizontal line within each box represents median; whiskers represent the range. The top horizontal line with asterisk demonstrates a significant difference between scores of registrars and foundation year two doctors; * = P < 0.05. FY1/2 = foundation year 1/2, SHO = senior house officer

**Figure 3:**
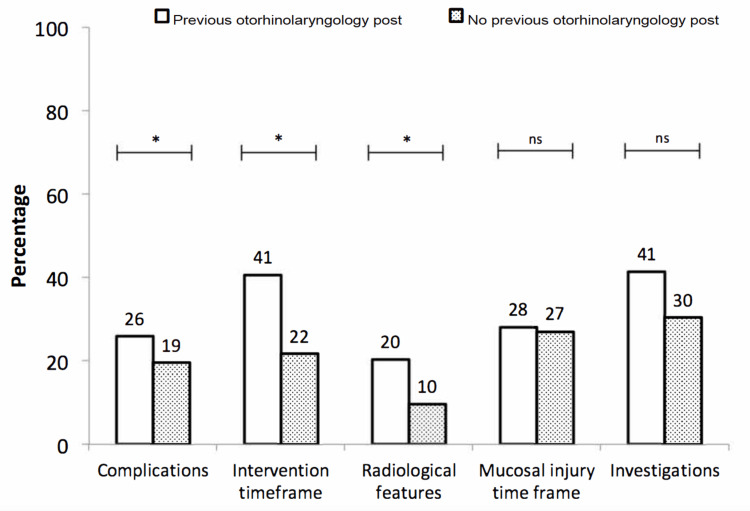
Comparison of percentages of correct responses to specific knowledge domains between participants with and without prior otorhinolaryngology experience Scores of two groups of participants with and without prior otorhinolaryngology experience are shown with clear and dotted bars respectively; the top horizontal lines with asterisks indicate the significance level of the difference in scores between the two groups for each domain; * = P < 0.05, ns = non-significant.

The median score out of 16 marks on the knowledge-based questionnaire was 18.8% (n=176, IQR=12.5-31.3%). The highest median score was 25%, which was scored by both registrars (n=37, IQR=18.8-37.5%) and consultants (n=18, IQR=10.9-34.5%). The remaining three grades of clinicians all scored a median of 18.8%: SHOs (n=68, IQR=12.5-31.3%), FY2s (n=45, IQR=12.5-28.1%), and FY1s (n=8, IQR=7.8-40.6%) (Figure 2). There was a difference in total percentage scores amongst the five clinician grades (P=0.0445), although narrowly within the 5% significance level. This may have been a chance finding due to multiple testing. Post hoc adjustment revealed a difference only between registrars and FY2 doctors (P<0.05). Participants that had previous otorhinolaryngology posts scored higher with a median of 31.3% (n=32, IQR=18.8-37.5%) compared to the rest of the participants (18.8%, n=144, IQR=12.5-31.3%). This difference was statistically significant (P=0.002). Participants with otorhinolaryngology experience scored statistically higher in questions relating to complications (P=0.0207), intervention timeframe (P=0.0246), and radiological features (P=0.0495). No significant difference was demonstrated in knowledge relating to the timeframe for mucosal damage (P=0.907) and investigations (P=0.0819) (Figure 3).

## Discussion

The majority of completed questionnaires were obtained from senior house officers. Only 18% of those surveyed were aware of an internal protocol for BBI. As only awareness of an internal protocol was assessed by means of a survey of a sample of clinicians, we cannot be certain whether the 11 participating hospitals actually had a protocol in place, only that a small proportion were aware of one. This is still a meaningful outcome measure as the existence of a local protocol may not be helpful if staff are not trained on accessing and using it. Houston et al. [7] surveyed 49 acute NHS hospitals in the UK on whether they had a guideline on ‘ingestion or inhalation of button battery or foreign body’. Of the 40 hospitals that responded, 28 (70%) had a guideline. Seven hospitals used TOXBASE® as their primary guidance [7]. We identified a high rate of routine screening for BBI by 70% of our respondents. This self-reported rate was higher than expected, given that poor screening for BBI was cited as a contributory factor in an NHS England inquiry into adverse incidents following BBI [5]. 

The median score on our knowledge-based questionnaire was 18.8%. Our study highlighted a number of areas of knowledge that required improvement. A considerable proportion of our study participants (38%) thought that the onset of severe mucosal injury would exceed two hours. Fourteen percent of respondents were unable to list a single complication. Only 13% correctly identified the need for a combination of neck, chest, and abdominal plain film radiographs. There was variation in plain film selection amongst our respondents as some chose to only image the neck (28%), chest (60%), or abdomen (28%). Houston et al. [7] also identified variation between hospitals in their BBI guidelines as only 33% recommended a combination of the chest, abdominal, and lateral neck soft tissue plain radiographs. The lack of consistency for imaging recommendations could lead to delays in the identification and management of BBI. A lack of understanding of the devastating complications, imaging requirements, and the timeframe for the onset of life-threatening complications may explain the inappropriate NCEPOD priority for removal of a button battery that was stated by 75% of our respondents. Similar findings were highlighted in the NHS England incident reporting data [5], which indicated that healthcare staff did not recognize the need for ingested button batteries to be treated as a medical emergency. Previous otorhinolaryngology experience was associated with higher scores (P=0.002) in our knowledge questionnaire. This indicates the merit of otorhinolaryngology training for emergency practitioners, which is not compulsory in the UK at present. The need for the emergency medicine curriculum to focus on otorhinolaryngology training is more pressing by the fact that otorhinolaryngology-related ED admissions have experienced a 14% annual rise [8]. A number of recent measures have aimed to address the educational deficiencies related to BBI, such as the publication of the first national UK guideline on management of BBI endorsed by ENT UK [7]; the formation of The European Society for Pediatric Gastroenterology Hepatology and Nutrition (ESPGHAN) task force for BBI, and a recent consensus statement on a BBI treatment algorithm [9]. These publications can aid NHS organizations to implement a standardized and consistent approach to assessment and management of BBI, and therefore enable them to fulfill the recommendations of the national patient safety alert [5].

Our study provided a regional snapshot of knowledge about the recognition and management of BBI of several emergency departments in the West Midlands, UK. The questionnaire was limited to a single region in the UK and therefore has limited generalizability. The absence of nurses and paramedics in the survey participants reduces the applicability of our findings to emergency departments as these allied professionals often have extended roles as emergency practitioners in the UK. The questionnaire was comprehensive but lacked validation; hence the reproducibility of responses may be hindered. The questionnaire was voluntary, and therefore it was prone to selection bias. The opportunistic distribution of electronic and hard copy questionnaires did not permit the evaluation of a response rate. Consequently, it is difficult to gauge how representative our findings are for the studied population. Further research is needed in order to evaluate the educational impact of recent UK guidelines and their integration into acute management pathways in the NHS.

## Conclusions

In view of the catastrophic nature of complications associated with BBI, a high index of suspicion must be maintained for any child presenting to the emergency department with a history or signs of an ingested foreign body. Our findings highlighted several areas of knowledge relating to the acute management of BBI that require improvement. The “golden hour” is accepted globally in trauma medicine, and this concept should be applied to the management of button batteries impacted in the esophagus. Wide dissemination of the recently published ENT UK and ESPGHAN clinical guidelines and local integration into acute management pathways is required to achieve a standardized and multi-disciplinary approach to BBI. Further research is needed to evaluate the impact of educational strategies on improving the knowledge base amongst frontline emergency practitioners.

## References

[1] Litovitz T, Whitaker N, Clark L (2010). Preventing battery ingestions: an analysis of 8648 cases. Pediatrics.

[2] Lin VY, Daniel SJ, Papsin BC (2004). Button batteries in the ear, nose and upper aerodigestive tract. Int J Pediatr Otorhinolaryngol.

[3] Litovitz T, Whitaker N, Clark L, White NC, Marsolek M (2010). Emerging battery-ingestion hazard: clinical implications. Pediatrics.

[4] Sethia R, Gibbs H, Jacobs IN, Reilly JS, Rhoades K, Jatana KR (2021). Current management of button battery injuries. Laryngoscope Investig Otolaryngol.

[5] (2021). Patient safety alert stage one: warning - risk of death and serious harm from delays in recognising and treating ingestion of button batteries. https://www.england.nhs.uk/publication/patient-safety-alert-risk-of-death-and-serious-harm-from-delays-in-recognising-and-treating-ingestion-of-button-batteries.

[6] (2021). The NCEPOD classification of intervention. https://www.ncepod.org.uk/classification.html.

[7] Houston R, Powell S, Jaffray B, Ball S (2021). Clinical guideline for retained button batteries. Arch Dis Child.

[8] (2021). Accident and emergency statistics: demand, performance and pressure - briefing paper number 6964. https://commonslibrary.parliament.uk/research-briefings/sn06964.

[9] Mubarak A, Benninga MA, Broekaert I (2021). Diagnosis, management, and prevention of button battery ingestion in childhood: a European Society for Paediatric Gastroenterology Hepatology and Nutrition position paper. J Pediatr Gastroenterol Nutr.

